# Supplementary material for: The impact of subject positioning on body composition assessments by air displacement plethysmography evaluated in a heterogeneous sample
Source: PLoS One. 2022 Apr 15;17(4):e0267089. doi: 10.1371/journal.pone.0267089 (PMC9012354; doi:10.1371/journal.pone.0267089)
Supplement: S1 Fig — On these schematic drawings of the front (left) and back (right) of the human body, the number displayed on, or next to, each body part gives its area as a percentage of the body surface area. Certain body parts are labeled by letters and their areas are listed below the schemes as a function of BMI. (The drawings were reproduced and modified with permission from the work of Cheah et al. [20]). (PDF) [file pone.0267089.s001.pdf]

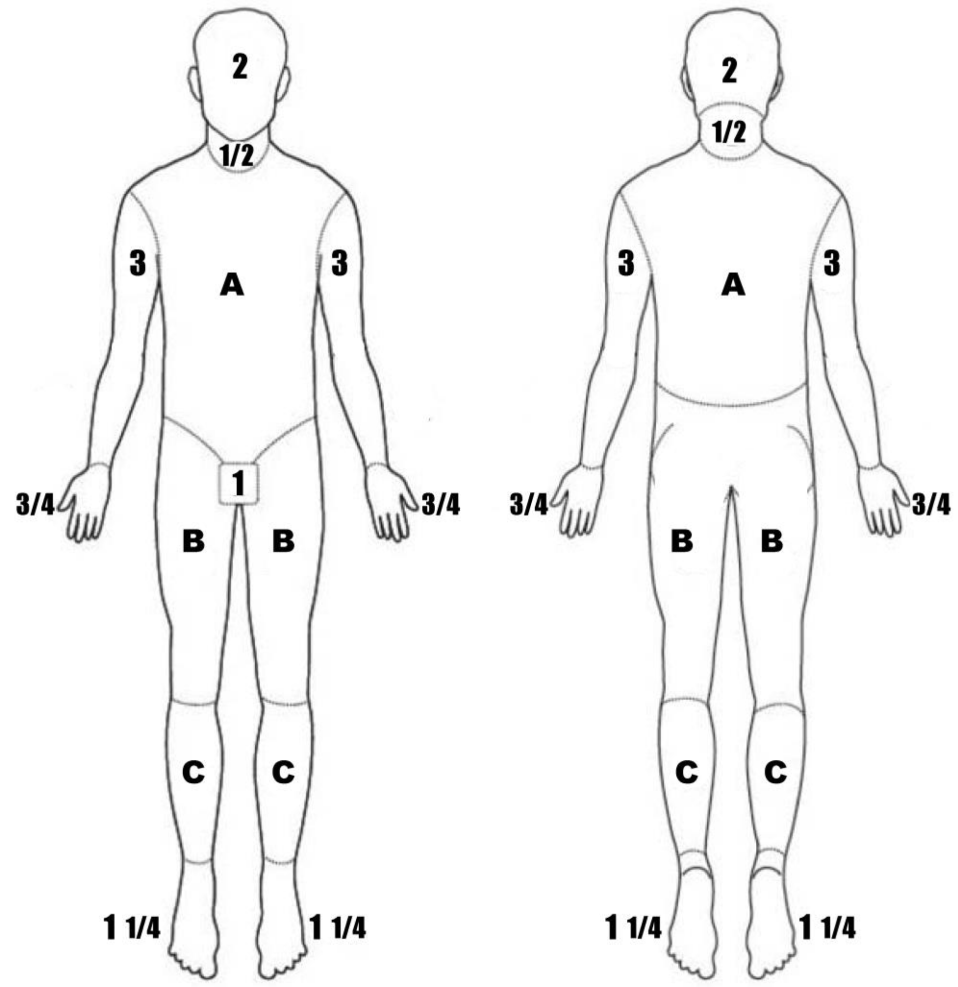

Area	BMI<30	30≤BMI≤39.9	BMI≥40
A = anterior / posterior trunk	17 1/2	20	22 1/2
B = half of one thigh	6 1/4	5 1/2	4 3/4
C = half of one lower leg	3 1/2	3	2 1/2

S1 Fig. The modified Lund-Browder chart that takes into account nutritional status [23]. On these schematic drawings of the front (left) and back (right) of the human body, the number displayed on, or next to, each body part gives its area as a percentage of the body surface area. Certain body parts are labeled by letters and their areas are listed below the schemes as a function of BMI. (The drawings were reproduced and modified with permission from the work of Cheah et al. [20].)
