# Supplementary material for: The impact of subject positioning on body composition assessments by air displacement plethysmography evaluated in a heterogeneous sample
Source: PLoS One. 2022 Apr 15;17(4):e0267089. doi: 10.1371/journal.pone.0267089 (PMC9012354; doi:10.1371/journal.pone.0267089)
Supplement: S2 Fig — The most common body surface charts used for evaluating burn patients: (A) the “Rule of Nines” [25], and (B) the Lund-Browder chart [24]. In each panel, shown are various body parts, viewed from the front (left scheme) and back (right scheme). The numbers displayed on, or next to, body parts express their areas as percentages of the body surface area. (The schematic drawings from this figure were reproduced with permission from the work of Cheah et al. [20]). (PDF) [file pone.0267089.s002.pdf]

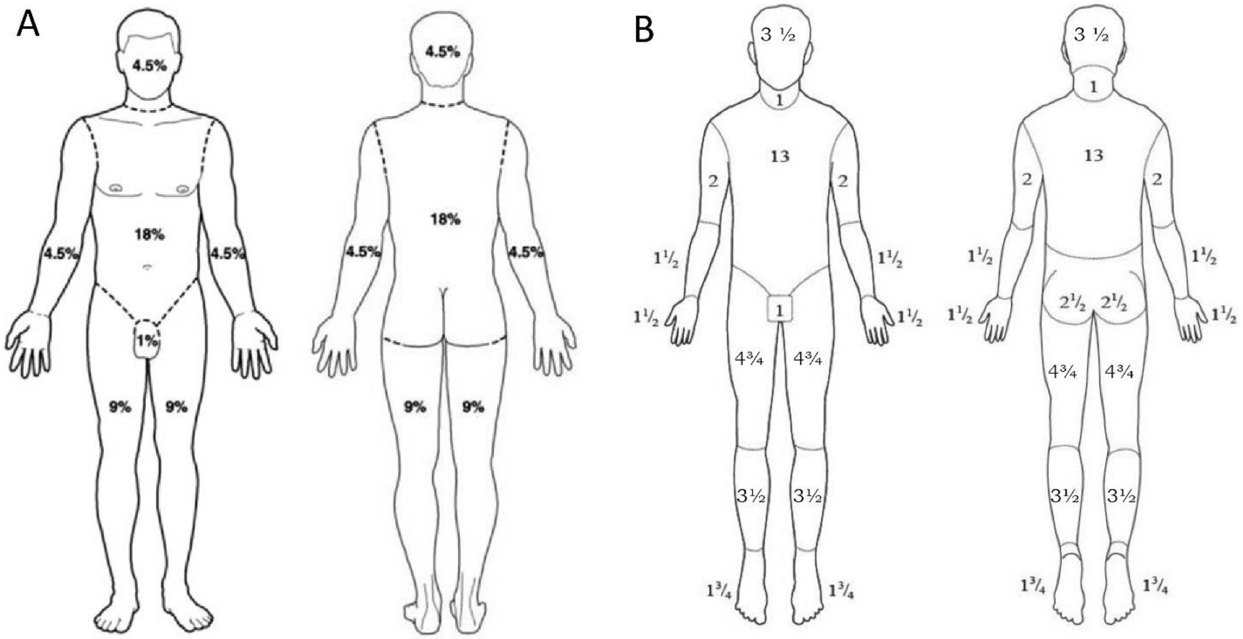

S2 Fig. The most common body surface charts used for evaluating burn patients: (A) the "Rule of Nines" [25], and (B) the Lund-Browder chart [24]. In each panel, shown are various body parts, viewed from the front (left scheme) and back (right scheme). The numbers displayed on, or next to, body parts express their areas as percentages of the body surface area. (The schematic drawings from this figure were reproduced with permission from the work of Cheah et al. [20].)
