# Supplementary material for: The impact of subject positioning on body composition assessments by air displacement plethysmography evaluated in a heterogeneous sample
Source: PLoS One. 2022 Apr 15;17(4):e0267089. doi: 10.1371/journal.pone.0267089 (PMC9012354; doi:10.1371/journal.pone.0267089)
Supplement: S3 Fig — The drawings represent the front and back of the body (left and right, respectively); letters label body parts whose surface areas are listed below the drawings, as percentages of the body surface area. (PDF) [file pone.0267089.s003.pdf]

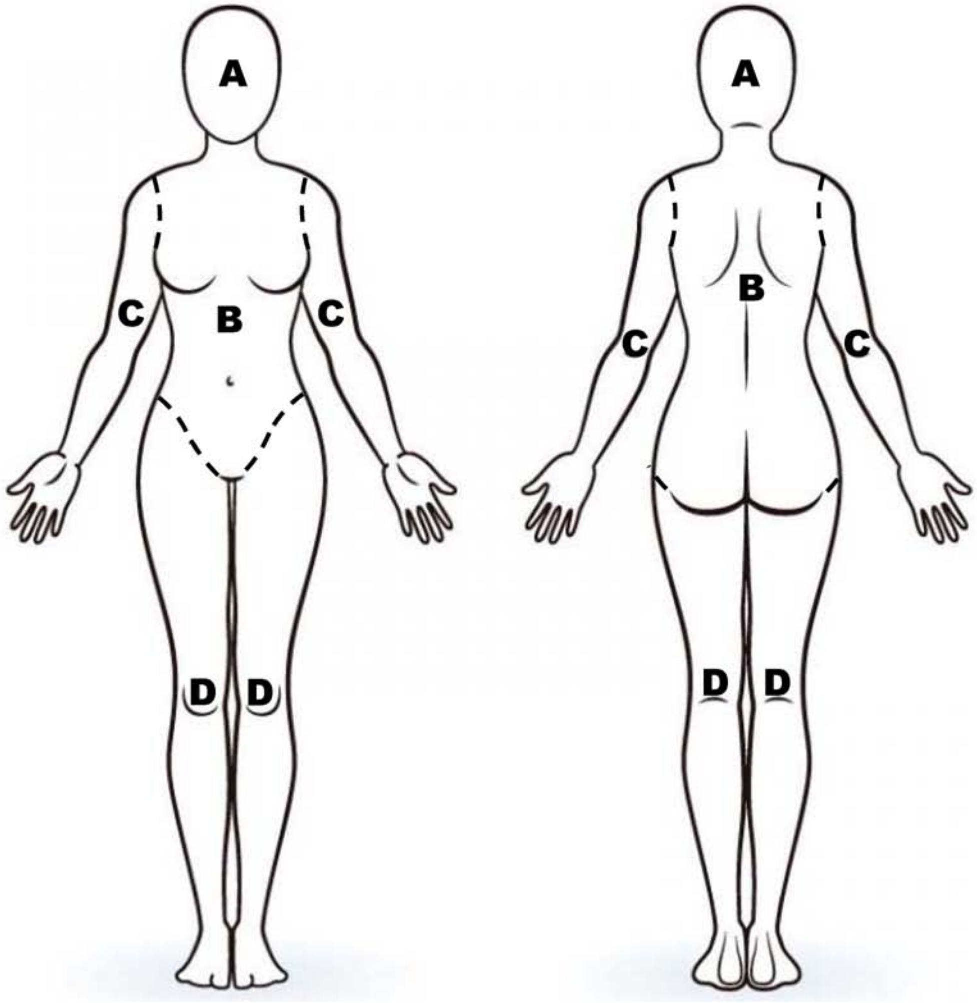

Area	18.5≤BMI≤24.9	25≤BMI≤29.9	30≤BMI≤34.9	BMI≥35
A	5	5	4.5	4.5
B	20	20	19.5	17.5
C	4	4	4	4.5
D	8.5	8.5	9	9.5

S3 Fig. The "Rule of Nines" for women, revised by taking into account their BMI [26]. The drawings represent the front and back of the body (left and right, respectively); letters label body parts whose surface areas are listed below the drawings, as percentages of the body surface area.
