# Supplementary material for: The impact of subject positioning on body composition assessments by air displacement plethysmography evaluated in a heterogeneous sample
Source: PLoS One. 2022 Apr 15;17(4):e0267089. doi: 10.1371/journal.pone.0267089 (PMC9012354; doi:10.1371/journal.pone.0267089)
Supplement: S4 Fig — The plots represent differences vs. means of BV (A), %BF (B), and FFM (C) obtained in relaxed position and compact position (upon correction). In each panel, the black solid line represents the bias, defined as the mean of the differences, whereas the black dashed lines delimit the 95% confidence interval (CI) of the bias. Red solid lines represent the 95% limits of agreement (the lower and upper limit of agreement, given by bias ± 1.96 × the standard deviation of the differences), whereas red dashed lines depict the corresponding 95% CI. Also, the equation of the regression line, the corresponding p-value, and the coefficient of determination (R2) are displayed on each plot. (PDF) [file pone.0267089.s004.pdf]

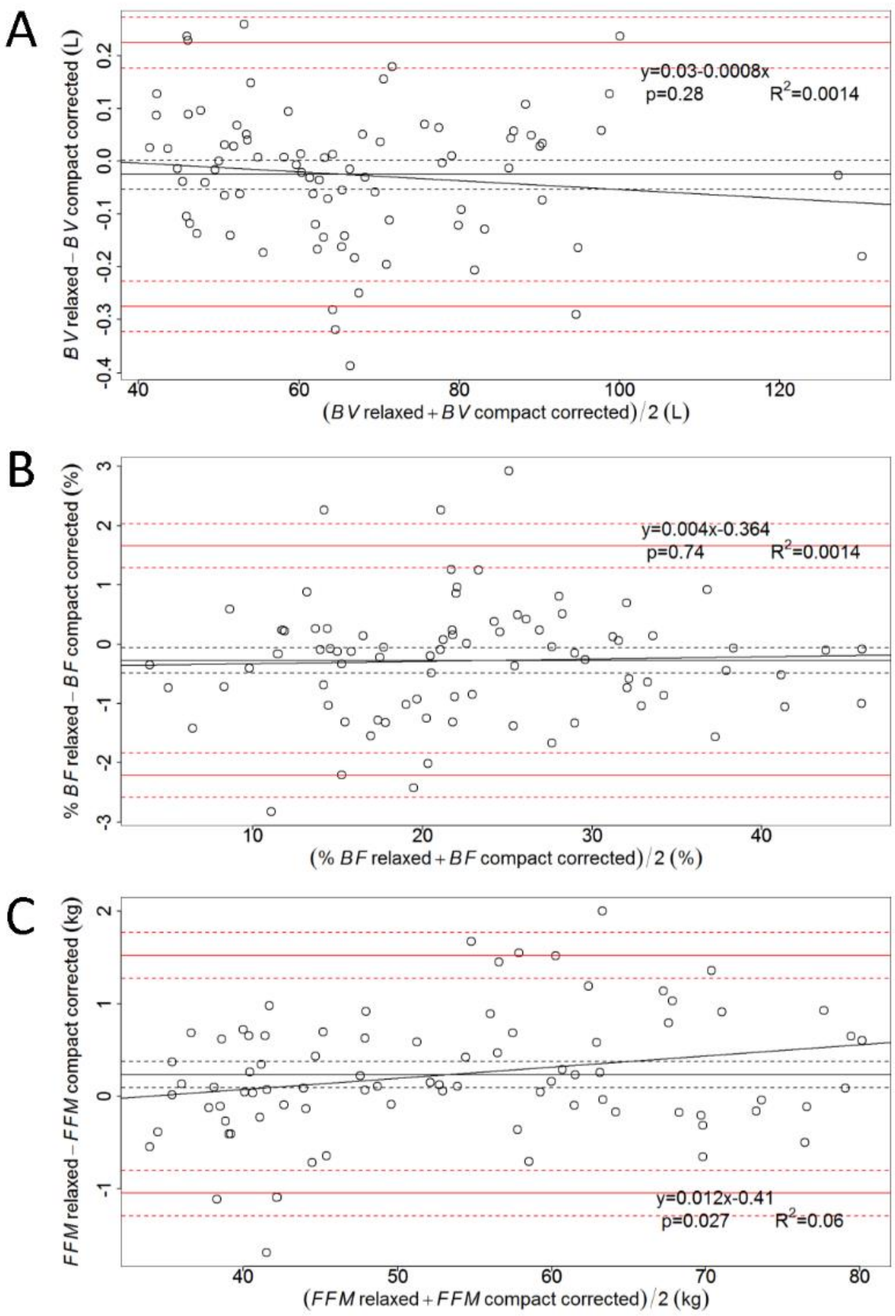

S4 Fig. Bland-Altman plots for *BV*, *%BF*, and *FFM*, obtained by using the Lund-Browder chart [24] to estimate the actual *SAA* associated to the compact position. The plots represent differences vs. means of *BV* (A), %*BF* (B), and *FFM* (C) obtained in relaxed position and compact position (upon correction)**.** The plots represent differences vs. means of *BV* (A), %*BF* (B), and *FFM* (C) obtained in relaxed position and compact position (upon correction)**.** In each

panel, the black solid line represents the bias, defined as the mean of the differences, whereas the black dashed lines delimit the 95% confidence interval (CI) of the bias. Red solid lines represent the 95% limits of agreement (the lower and upper limit of agreement, given by bias $\pm$ 1.96 $\times$ the standard deviation of the differences), whereas red dashed lines depict the corresponding 95% CI. Also, the equation of the regression line, the corresponding p-value, and the coefficient of determination (R^2) are displayed on each plot.
