# Supplementary material for: The impact of subject positioning on body composition assessments by air displacement plethysmography evaluated in a heterogeneous sample
Source: PLoS One. 2022 Apr 15;17(4):e0267089. doi: 10.1371/journal.pone.0267089 (PMC9012354; doi:10.1371/journal.pone.0267089)
Supplement: S6 Fig — The plots represent differences vs. means of BV (A), %BF (B), and FFM (C) obtained in relaxed position and compact position (upon correction). Notations are explained in the caption of S4 Fig. (PDF) [file pone.0267089.s006.pdf]

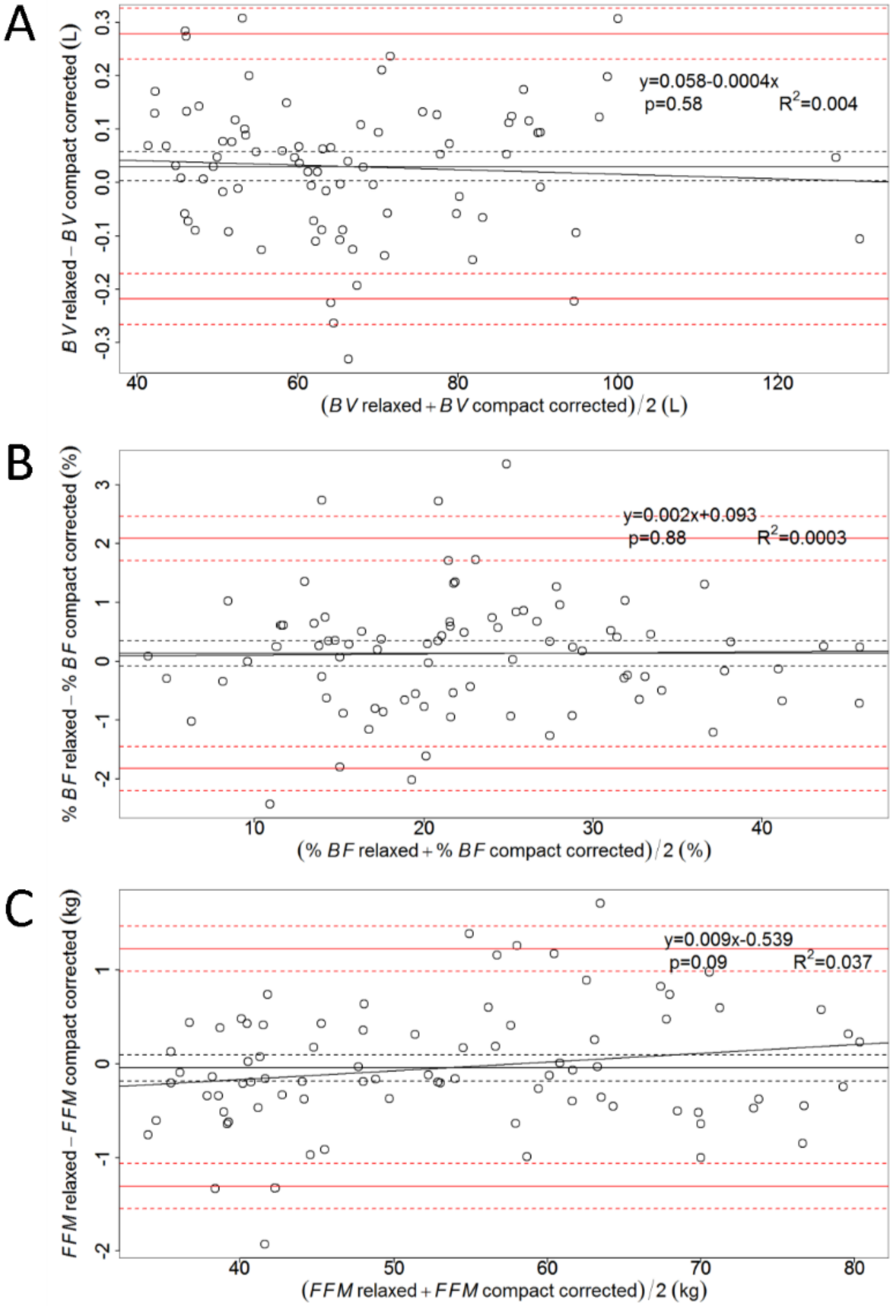

S6 Fig. Bland-Altman plots for *BV*, *%BF*, and *FFM*, generated by applying the "Rule of Nines" [25] to estimate the actual *SAA* corresponding to the compact position. The plots represent differences vs. means of *BV* (A), %*BF* (B), and *FFM* (C) obtained in relaxed position and compact position (upon correction)**.** Notations are explained in the caption of S4 Fig.
