# Supplementary material for: The impact of subject positioning on body composition assessments by air displacement plethysmography evaluated in a heterogeneous sample
Source: PLoS One. 2022 Apr 15;17(4):e0267089. doi: 10.1371/journal.pone.0267089 (PMC9012354; doi:10.1371/journal.pone.0267089)
Supplement: S7 Fig — The plots represent differences (relaxed-compact) vs. means of BVr (A), BV (B), %BF (C), and FFM (D). Notations are explained in the caption of S4 Fig. (PDF) [file pone.0267089.s007.pdf]

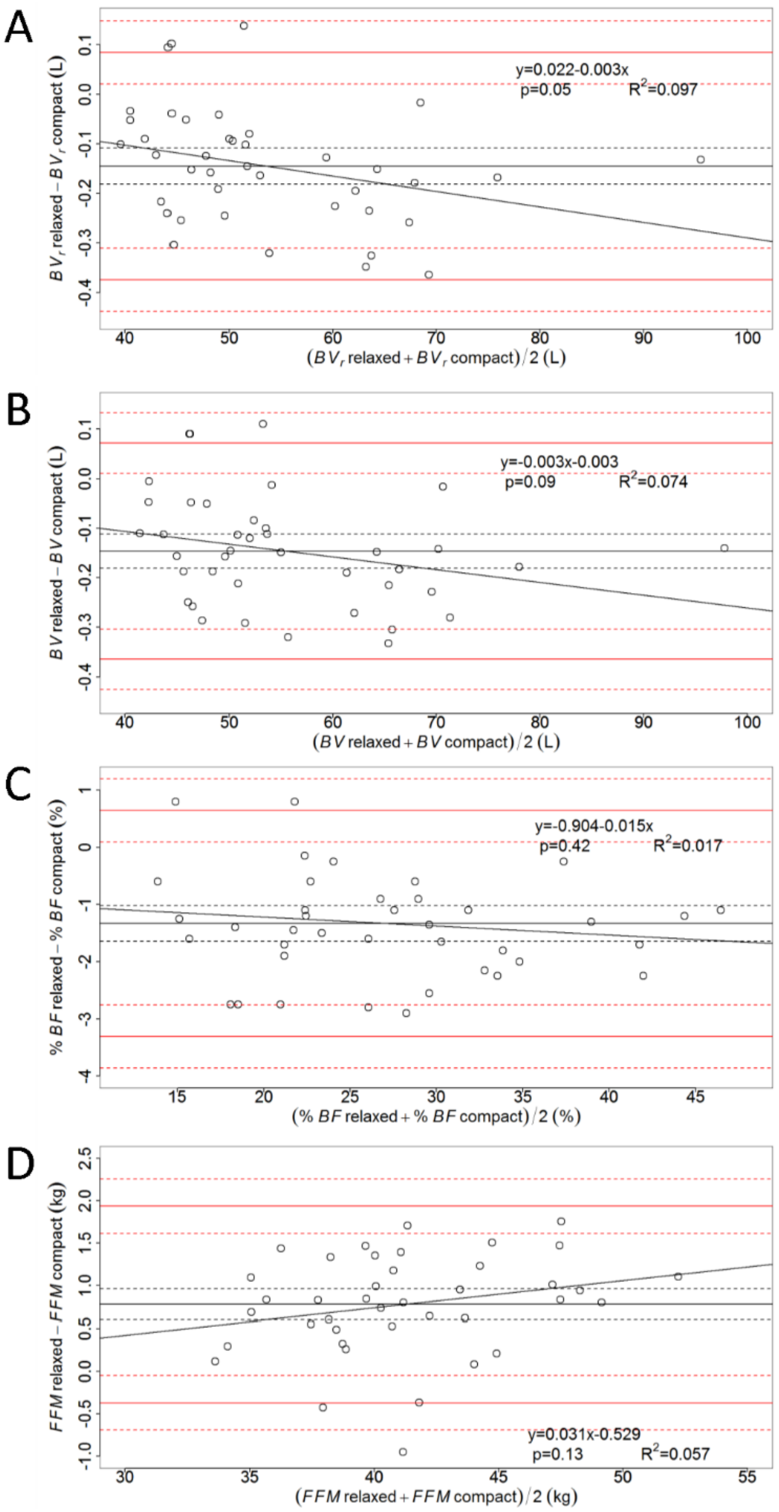

S7 Fig. Bland-Altman plots of *BVr*, *BV*, *%BF*, and *FFM* of female subjects. The plots represent differences (relaxed-compact) vs. means of *BVr* (A), *BV* (B), *%BF* (C), and *FFM* (D). Notations are explained in the caption of S4 Fig.
