# Supplementary material for: The impact of subject positioning on body composition assessments by air displacement plethysmography evaluated in a heterogeneous sample
Source: PLoS One. 2022 Apr 15;17(4):e0267089. doi: 10.1371/journal.pone.0267089 (PMC9012354; doi:10.1371/journal.pone.0267089)
Supplement: Attachment — Submitted filename: PLoS Comments to the Authors.docx [file pone.0267089.s017.docx]

Comments to the Authors.

Thank you for your in-depth investigation of factors influencing calculation of the SAA for adults undergoing ADP assessments! Your findings and insights provide great depth of understanding as to why body positioning within the BodPod chamber is critical – especially for serial assessments of body composition.

To conform to the conventions and grammar of the English language, this reviewer suggests enlisting services of a native English speaker to help clarify your otherwise well-written report. Suggestions for both grammar, clarity, and content are below. If the journal provided editing services, this list would be much shorter.

Line 48 – “compartment” should be “component”

Line 67 – by “softer” do you mean “less dense”? Please clarify

Line 70 - change to read “as well as is a …”

Line 72 - change to read “hair be completely…”

Line 84 – this reviewer did not see the definition of BSA and TGV prior to their use in the BV formula

Line 113 – change to read “positions that concern”

Line 128 – use “lower and upper bounds” instead of “range” in table title

Line 125 – move “, including scale calibration,” to follow “procedure in line 134

Line 138 – indicate if participants were barefoot for standing height

Line 141 – change to read “wore dry, minimal….”

Line 153 – be sure the sequence of posture in Fig 1 matches specifics of the figure title.

Line 154 – would be helpful to identify if participants exited the chamber between trials in Tucker’s study.

Line 158 – even though you are most interested in SAA, indicated if TGV was estimated or measured? If estimated, mention that as a limitation.

Line 163 – change to read “In the compact…”

Lines 185-189 – is “VTG” supposed to represent “TGV”? The latter is a more common term, Be sure to define the term prior to its use

Line 193 – insert a space before ±

Line 225 – needs to be a continuation of line 224. Looks like there was a line feed

Line 255 – references to SI figures no longer follows ascending numbering sequence. Reference to S Fig 3 occurs on line 170, but no reference to S figures 4 -6 found prior to reference to S7 figure. Reference to S44-S6 comes on line 274. See below for comments regarding clarity of the figure titles/legends.

Line 277 – Is it not more appropriate to indicate that it is “The results from this study are in accord…”?

Line 282 – consider changing to read “positions that concern the fraction…”

Line 293 – consider using “Peeters and colleagues [18]” instead of “ref [18]” as the subject of your sentence

Line 303 – change “ref [13]” to the first author’s name followed by [13].

Line 307 – change to read “in close vicinity to the”

Line 313 – consider clarifying who/what is being referred to aby “Both of them”. It seems you are referring to techniques, but it is not clear as written.

Line 316 – by “This study” are you referring to “The present study” meaning your study? Not clear as written.

This reviewer did not see a reference to S13 or S16.

In reference list, check format for journal title for #19. It seems to be the only one that spells out the complete journal title

My primary concern is with the figure titles/descriptions of the Bland and Altman SI plots using both red and black line colors. Some of the SI figures have two solid red lines with 95% LOA dashed lines, but no indication I found as to what they were indicating. While this suggestion may be perceived by the authors as adding redundancy, it would clarify some confusion for readers like me who have extensive experience with Bland & Altman plots. It would also help with the concept about each figure being able to stand alone.

Another concern was the inability to understand “the numbers displayed …” aspect of the title for SI Fig. Providing an example would go a long way in providing clarity of what you are trying to say.
